# A Comprehensive Framework to Optimize Short-Term Experiences in Global Health (STEGH)

**DOI:** 10.1186/s12992-019-0469-7

**Published:** 2019-04-02

**Authors:** Shivani Shah, Henry C. Lin, Lawrence C. Loh

**Affiliations:** 1 Schulich School of Medicine and Dentistry, University of Western Ontario, London, ON Canada; 2 Oregon Health Sciences University, Portland, Ore Canada; 3 Brooklyn, NY USA; 4 Dalla Lana School of Public Health, University of Toronto, Toronto, ON M5T 3M7 Canada

**Keywords:** Global health, Ethics, Volunteering, Medical mission, Motivations

## Abstract

Increasing demand for Short-term Experiences in Global Health (STEGH), particularly among medical trainees, has seen a growth in programming that brings participants from high-income countries to low and middle-income settings in order to engage in service, teaching or research activities. Historically the domain of faith-based organizations conducting “missions”, STEGH are now offered by diverse groups including academic institutions, non-profit organizations, and the private sector, either as dedicated for-profits or through corporate social responsibility arms.

The growing popularity of STEGH has resulted in concerns about their negative impacts on host communities. Traditional STEGH are often crafted with little or no input from host community leaders, and this results in activities that do not address locally identified priorities. Other concerns include culturally incongruent programming and the creation of parallel systems that disrupt established local services and redirect scarce local resources, which fosters dependency instead of building capacity. One concern specific to trainees also includes trainee provision of services beyond their scope and training level.

To address these concerns, this paper presents a comprehensive framework that aims to categorize promising interventions that might promote greater responsibility in STEGH. Based on the micro-meso-macro framework, this paper proposes various interventions as incentives and disincentives to be deployed at the individual, program, and societal levels to promote greater responsibility in STEGH. Deployed altogether, the interventions contemplated by this framework would foster the optimal context  required to encourage responsibility, minimize harms, and optimize host community outcomes for STEGH.

## Background

Short term experiences in global health (STEGH) have grown in popularity in recent years, particularly among trainees and learners. [1]. STEGH typically involve volunteer and learners from high income countries (HICs) travelling to settings in low and middle-income countries (LMIC) typically to provide service, teach, or conduct research [2–4]. Such efforts vary in length from one to several weeks and are offered by many different types of organizations including academic institutions, faith-based organizations, non-profit organizations, and the private sector, including for-profit organizations and corporate social responsibility arms [4, 5].

A 2005 estimate of the value of United States volunteer time spent abroad was estimated at $2.92 billion, with 10 million volunteers participating in STEGH. [3–5] Specific to medical students, participation rates in STEGH increased from 6% of matriculating medical students in 1978 to 32% in 2008, and latest data from the American Association of Medical Colleges suggests that 43.2% of currently enrolled American medical students have participated in some type of STEGH [2].

In parallel with the increasing popularity of STEGH, concerns have risen around potential negative impacts on host communities and the need to be thoughtful and responsible in the conduct of STEGH [6, 7]. A comprehensive review of over 230 studies on STEGH identified unsustainability of outcomes, inappropriate allocation of scarce resources, paucity of data and evaluation, cost-effectiveness, unfamiliarity and a lack of sensitivity to local needs and culture, practicing beyond scope, complications, and limitations in achieving an appropriate standard of practice as defined negative impacts. [8] Other concerns described in literature include perpetuation of power differentials, the cultivation of dependence on visiting foreign-service providers, and disruption to local health and social service systems within host communities [9–11]. Finally, STEGH are also often criticized for overlooking evaluation and impact assessments. [3]

This paper proposes a categorical framework of interventions which are intended to create the incentives and conditions necessary to promote a baseline of responsibility in the conduct of STEGH, irrespective of considerations such as the affiliation of a sending organization or the professional level of training of participants. [12] Tied to accomplishing a foundational set of guiding ethical principles, such as those proposed by the WEIGHT guidelines, [8] the suite of interventions considered for deployment in this framework are categorized as individual, programmatic, and societal and contextualized within sending and receiving countries as well as internationally.

This categorization is based on a micro-meso-macro framework drawn in public health literature around health promotion and behaviour change, as was done most notably in tobacco control. [13, 14] Literature identifies that education alone, targeted at individual smokers, would have limited impact in addressing smoking behaviours at a population level. Today’s tobacco control success arose from a comprehensive suite of programmatic and policy changes to context more broadly that discouraged tobacco use and encouraging cessation. [15]

Promoting a responsibility and outcomes-focused lens through a similar comprehensive approach could minimize harms and optimize the benefits of STEGH for hosting communities. [4] This debate paper thus aims to deploy the micro-meso-macro framework in identifying a similar set of interventions for debate and discussion that, if effectively deployed, might provide the impetus to reshape how STEGH are conducted.

## Individual level interventions

Drawing on the tobacco control example,   the individual level of this framework is aimed at changing mindsets. For tobacco users, this entailed addressing motivations and behavioural factors, including their perceived barriers to quitting, through provision of cessation services [16]. Within the realm of STEGH, targeting individual volunteers is about changing perspectives so that participants recognize their participation as a privilege and not a right. To encourage participant behaviour change, individual level interventions have two broad goals in line with described principles: firstly, to encourage volunteers to work under the leadership and direction of host community partners, and seek out programs that do so, and secondly, to ensure that volunteers are adequately trained, prepared, and deployed, with a focus on understanding the importance of how to humbly and critically reflect on their experience at all stages, consider their intentions in volunteering, and identify blind-spots. [1, 10].

Given these two areas of focus and the reality that many volunteers are learners, potential approaches that may be promoted at the individual level should include but are not limited to:

- Educating volunteers to think less about *saving the world* and instead focus on desired outcomes identified by host communities and working with local leaders to critically consider the underlying complexity of factors that drive those challenges; [1, 7, 9, 10]
- Encouraging careful self-assessment and awareness of one’s level of training and limits to guide their involvement. Stated simply, the golden rule in this situation is to ensure that volunteers only participate in activities and providing care for which they have received training; if one cannot provide that service at home, it is likely inappropriate to provide that abroad. [9, 10]
- Taking the opportunity to explore considerations, as possible, with relevant expatriate communities in home country prior to departure, to develop cultural sensitivity and related skills. [1, 7, 9, 10]
- Not participating in “one and done” stand alone efforts, but instead joining established programs that incorporate ongoing work. [1, 10]
- Considering alternatives to volunteering abroad in order to support global health and development efforts, with a recognition that addressing circumstances in one’s own home country that drive global inequities may have greater impact than working directly with the communities affected by those inequities. [16]

As with tobacco cessation, this framework recognizes the public health truism that changes in individual behaviour will likely not occur through education alone, and that educated volunteers would still require appropriate supports to shift the paradigm on how STEGH are conducted [17]. However, encouraging individual volunteers to adopt the described values above represents one contribution in reaching the overall goal of greater responsibility in STEGH.

## Program level interventions

To augment individual level changes, targeted interventions should aim to also change how programs are conducted. At the program level, we are proposing that organizations that offer STEGH deploy strategies to ensure that the programs are conducted responsibly. Drawing again on our parallel with tobacco, this meant monitoring tobacco use and providing specific incentives and disincentives to support the educational messages given around tobacco use [15]. In a similar manner, targeted incentives and disincentives should be employed to ensure that STEGH programs adhere to guidelines calling for responsible planning, conduct, follow-up, and evaluation. Broad categories include managing expectations, focusing on capacity building, fostering collaboration and partnership, rigorous volunteer selection, effective participant preparation, training, and debrief, and monitoring and evaluation. [1, 7–10, 12]

### Managing expectations

Programs that conduct STEGH should aim to appropriately manage the expectations of participants and host communities through a clearly articulated vision inculcating responsibility [10, 18]. This would include, among various considerations:

- Paradigm shift: Avoid promoting program goals as “fixing or saving the world”; instead encouraging humility and a focus on achieving lasting outcomes on priorities identified by community leadership [10, 18]
- Clear guidelines: Developing guidelines for program planning that support this shift in focus, ensuring host community impact is prioritized over participant self-advancement [10, 18, 19].
- Raising awareness: Deploying education and awareness campaigns via popular and social media that promote responsible participation in STEGH, targeted at key STEGH stakeholders such as funders, host countries, volunteers, and sending organizations. In keeping with the parallel around tobacco, literature demonstrates the effectiveness of such targeted communications strategies on knowledge and behaviour [20].

Such interventions might contribute to the overall strategy by setting the context and painting a realistic picture of the work for STEGH participants. Setting this context will allow those of a contrary mindset to reconsider their participation, while ensuring those who are genuinely committed to responsible participation receive affirmation. Raising awareness among stakeholders will similarly increase the likelihood that these stakeholders, particularly host community leaders and funders, expect a certain level of focus on outcomes and community leadership from programs as a condition of winning support.

### Focus on capacity building

Programs that conduct STEGH should aim to shift the focus of their work away from direct service delivery and move towards capacity building and sustainable development. Many of the criticisms around STEGH have highlighted the dependence and service perturbations created in host communities by visiting groups providing services. Switching the focus of efforts to capacity building around local priorities will help resolve this by ensuring that local health and social services are not disrupted by volunteer STEGH, preventing reorientation of services and the development of dependence. [1, 19]

Any move to capacity building, however, must be undertaken with the aim of engaging qualified volunteers in such efforts, and existing programs that do decide to transition would need to carefully manage these changes to mitigate potential impacts from any withdrawal of visiting services. [1, 7, 9, 10]

#### Foster collaboration and partnership

STEGH programs should aim to collaborate with other visiting programs that operate in the same local region, and also with representative host community leadership. One potential model could include the creation of a coalition among stakeholders with the aim of eliminating duplication, redundancy, and mixed-messaging in visitor activities while ensuring that there is local direction on priority projects that integrates with the established community efforts. [19–22]

#### Rigorous volunteer selection

Programs should also carefully ensure that their selection processes recruit volunteers with appropriate skills and ability that match the identified needs and projects being undertaken in the host community [1, 10]. Volunteers should also be selected not only for their skills, but also for the potential to complete needed preparatory training, critical thinking ability, cultural sensitivity, and insight around the privilege associated with their participation. [10] Implementing a rigorous selection process with clearly defined criteria and an understanding of the desired outcome of the project is crucial.

#### Effective participant preparation, training, and debrief

Pre-departure training might seem foundational to any STEGH experience, but one research study found great discrepancy among the 17 Canadian medical schools in their pre-departure training for STEGH, which varied from 30 min to 30 h. [2] Similarly, the classical focus of pre-departure training on participant safety and objectives fails to incorporate a broader lens on responsibility and community impacts that is proposed in many guiding principles. [1, 9, 10, 18]

Pre-departure training should be broadened in focus to target the development of hard skills necessary for the project (e.g. research methods, program planning) as well as soft skills needed to mitigate potential conflicts and misunderstandings (cultural sensitivity, ethics, consensus/team building). Testing should be conducted to ensure that concepts are learned and that participants are ready, and there should be an expectation that participants will be held back if they do not demonstrate required competencies. [21, 23, 24]

Post-return debriefing sessions should also be implemented as an evaluation and feedback tool to identify program issues and support continuous quality improvement. [10]

#### Monitoring and evaluation

Monitoring and evaluation must happen in two broad forms to encourage programs to include these responsible elements. Firstly, programs themselves should ensure that regular monitoring and evaluation is incorporated into their processes. Linked to the priorities identified by the host community leadership, programs will need to clearly define the impact of the work currently being done towards achieving locally identified priorities. Robust systems should track trends, needs, and areas for improvement that can inform planning and discussions with the host community. [9]

Secondly, programs might be monitored or certified by objective third party observers, such as professional associations or regulators. Using a benchmark of expected program elements around responsibility (e.g. “to what extent do your activities integrate with local community efforts”, or “how do your outcomes meet locally identified priorities”) such observers could identify exemplars of responsible engagement, while also highlighting deficient practices among other organizations. Other potential tracking mechanisms could include a seal or certification of responsibility, direct third-party audit, databases that track any and all reports of harms associated with STEGH, and awards to highlight models of responsibility. The use of such surveillance data and enforcement parallels evidence-based practice from various tobacco control efforts, such as tracking vendors, restricting outlet density, and enforcement of regulations controlling sales to minors. [25]

## Societal level interventions

The societal level of the framework captures interventions that aim to change context and social norms. Considering tobacco, this meant denormalizing tobacco and addressing the way society saw its use, through interventions like taxation and public place of use restrictions. [15, 25] Similarly, for STEGH, interventions must be deployed to, stated simply, make it “uncool” to go abroad with a program that does not meet a specific responsibility standard. Appropriately implemented, this means setting up contexts that provide incentives for responsibility in STEGH and disincentives for harmful or ineffective practices, for volunteers who choose to participate, sending organizations that plan specific programs, and communities that host STEGH.

By and large, these interventions will typically involve the deployment of policy changes internationally and in other settings at institutions/organizations, professional associations, licensing bodies, and government agencies and regulators.

### Context change in sending countries

#### Institutional policy changes

Policy changes in institutions can incentivize responsibility in STEGH. This can include, for example:

- Collaborating with academic institutions to change various admissions policies: At present, experiences abroad are generally seen positively for the purposes of admission to healthcare professional schools. Changing these policies to flag STEGH on a CV for interview or further follow-up as to whether the practices were ethical and conducted with responsibility in mind would act as an incentive that would drive students towards more responsible participation and programs. A certification program for responsible STEGH could see accreditation used as a proxy for the purposes of this incentive. [26]
- Changes to student / learner policies: Sending institutions can implement codes of conduct tied to disciplinary action (e.g. remediation, or reprimand on transcript) as an incentive for responsible participation on the part of students. This regime should apply regardless of whether learners participate in a school-sanctioned or external STEGH. Such enforcement parallels similar activities in tobacco control visited upon places of use or retailer regulations. [25]
- Changes to institutional policies: Increasing the scrutiny of student groups and affiliated organizations that conduct STEGH can help to uncover potential poor practices and also highlight responsibly conducted programs that could be emulated. From tobacco control literature, this parallels work done to highlight unethical practices associated with Big Tobacco that aimed to incentivize change. [27]

#### Regulatory and professional association changes

Laws and legislation in sending countries could be passed to encourage responsibility in practice, such that individuals who cause harm in their overseas volunteer activities are liable to prosecution in their home country for any instances that related to malpractice or unprofessional behaviour. This would parallel similar legislation that permits prosecution for acts such as child sex tourism at home, regardless of whether the crime occurred outside of the volunteer’s home jurisdiction. [28]

Regulatory colleges or professional associations could also facilitate inquiries of this nature through disciplinary committees; literature has shown that such disciplinary processes are effective at changing provider behaviour. [24, 29]

Insurance policies for providers who participate in STEGH can also include terms and conditions that invalidate the policy should a participant, or trainee under their supervision, practices out of scope or irresponsibly. Literature is clear that insurance rules are effective at changing provider behaviour. [30]

Finally, litigation undertaken by those harmed abroad by STEGH, against participants in their home jurisdiction, could also act as a deterrent, provided those bringing the action are appropriately empowered and resourced. [31]

#### Context change in host countries

Besides entry visas and certification, other considerations for host countries also include policy and regulation (e.g. institutional codes of conduct for visiting volunteers; governmental laws against malpractice, etc.) and the enforcement of such laws by governments of host countries. Host countries should be resources and empowered to strengthen their policies and laws and monitor, direct, and coordinate STEGH groups within their country to better align the work of STEGH with local systems and priorities. [10, 19, 31]

#### Visa requirements

Host country governments should be assisted in reviewing their visa requirements and classes to allow effective tracking of short-term volunteers. For example, the creation of a short-term visa class would allow monitoring and could require various prerequisites prior to issuance (e.g. assessment of skills, completion of a pre-departure module developed by the host country on culture / etc.). This might ensure that participants are better informed as they participate. Adherence to visa policy parallels similar public health programs which demonstrate that mandatory education associated with licencing of tobacco vendors reduces irresponsible practice of selling to minors. [32]

Such visas could be issued on the basis that participants have the appropriate licensure and are registered in their regulatory college of training (e.g. Royal College of Physicians and Surgeons in Ontario) before embarking on a STEGH, though host countries should also be empowered to enforce the required provisions of their own domestic licensure processes. [31]

#### Local guidelines and codes of conduct

STEGH participants should follow any relevant codes of conduct and laws of the host country, ensuring that their practice is in line with local priorities and expectations. [10, 19] Supporting host communities in the development of codes of conduct will help ensure that participants are aware of their specific roles and responsibilities and can aid in outlining the penalties of non-compliance. [19] In particular, for service-oriented trips, host countries should also be empowered and resourced to ensure that visitors are adhering to any protocols and guidelines that outline best practices and enforce any noncompliance.

#### Certification

Participants should not rely on licensure from their home country to justify their activities in a host country. [31] However, to ensure that participants have the intention of strengthening and empowering local communities, host countries or international bodies might consider establishing a new certification requirement and/or category specifically for entering a country for the purpose of a STEGH. (e.g. registered humanitarian.) In this manner, regulatory colleges could create a special certification class for STEGH that provides that if someone can demonstrate competence in their home country, that they can receive certification within the host country. This system could also track certified volunteers and programs for continued improvement and monitoring of outcomes and could be linked to a similar program as described at the “program” level to broadly monitor programs. Students on such experiences would still be expected to participate under the supervision of a preceptor that is properly licensed in-country. [31]

## Context change at the international level

### Declarations

At an international level, aspirational standards and declarations should be set to manage expectations for host and receiving countries. Much like the Framework Convention for Tobacco Control, [33] sending and receiving countries willing to participate in STEGH should encourage the development and ratification of an international declaration to set the tone for all interventions and foundational standards pertaining to STEGH. Flowing from that declaration and in light of existing recommendations from leading organizations such as the World Medical Association among others [34, 35] an international set of guidelines and/or code of ethics can be developed as a schematic for sending and host country leaders to regulate what can and cannot be done by STEGH participants. The caveat is that while a declaration and international guidelines will raise the profile of responsibility in STEGH worldwide, there are limits to the enforceability of international declarations, as evidenced by contemporary climate change declarations and the United Nations Declaration of Human Rights.

### Protocols and guidelines

International forums can serve as the catalyst for outlining the principles around best practices and guidelines for stakeholders that will support change at all three levels. In particular, a set of principles around responsibility should extend beyond simple clinical guidelines or service protocols with the intention of providing overarching ethical standards that underpin any STEGH activities undertaken by visiting participants. [1, 10, 35] Related to this would also be a commitment to update these as needed based on ongoing research in key ethical considerations around STEGH.

### Resources

Intergovernmental organisations should also provide resources towards implementing a broad strategy to inculcate responsibility in STEGH, and support continued meetings and conference that will drive conversations on the best way to turn the tide around many of the concerns expressed to date.

## Discussion

It is important to highlight that the interventions described within this framework all have merit in creating the contexts needed to move the needle on STEGH. As described, this work parallels tobacco control efforts, in trying to change behaviour through individual, programmatic, and societal-level interventions. [15, 20, 25, 27, 32, 33]

This paper suggests that, if successfully and effectively implemented, this framework would drive greater responsibility in STEGH. By changing contexts and encouraging greater responsibility through societal-level incentives, programs will be required to change and adhere to specific expectations, which in turn would make it easier for individuals who are aware of the importance of this topic to go abroad in a responsible manner. This echoes tobacco efforts that targeted structure, contexts, and individuals to make the healthy choice of not smoking the easy choice. [13] For STEGH, the aim is to ensure that the paradigm shifts to ensuring that the needs and priorities of host communities come first in any volunteer endeavour, with incentives to support volunteers with the right mindset and programs that have the appropriate processes and focus in place, and disincentives in place for those without the requisite skills or understanding. [10]

This framework also has a strength in the monitoring and evaluation aspects that will allow characterization of the extent and nature of the phenomenon. Monitoring on the part of a regulator or association would allow examination of data on the current impacts of existing practices and identify successful models and means for improvements. Over time, this would drive the creation of best practices in program processes (e.g. around effective participant selection, appropriate pre-departure training, or community partnership) that could support the deployment of these elements in programs across many sectors.

The framework also encourages the fostering of dialogue across various settings to ensure a commitment to impact and effectiveness and disincentivize “irresponsibly conducted” STEGH through tactics such as litigation or denormalization. [30, 31, 36]

The desired outcome of these efforts is that the paradigm would eventually shift to ensuring that host communities truly benefit from STEGH efforts in a way that they do not presently, and that STEGH would be directed by local leadership as a meaningful contributor to ongoing efforts, rather than developing dependence and disrupting local systems already in place. [10, 19, 37] In the long run, the use of skilled, prepared volunteers to develop sustainable solutions could assist development efforts and drive the building of lasting capacity and resilience. [37]

There are certainly challenges with this proposed framework, largely around implementation. Foremost is determining who will lead the implementation of aspects of this framework. This is compounded by the need to deploy different strategies in various settings, which will require any leading organization to take on a convenor role rather than providing support to all the tactics themselves. Any such organization will need to figure out how to prioritize and raise the importance of STEGH among key bodies (e.g. regulatory colleges, associations, and international organizations) as well as how best to engage and/or regulate groups that conduct STEGH.

Another challenge is that current variability in the ethical conduct of STEGH is due to existing contexts and incentives that make it easy to ignore guiding principles. Stakeholders should thus be carefully consulted around which of the framework elements should be first implemented to create appropriate incentives that will encourage the adoption of other framework elements in the future. One other challenge that arises upon full implementation of this framework would be how to respond to unskilled students and others who may wish to participate in a STEGH but do not match the required skillset. It will certainly be difficult, especially in the initial phases, to track or monitor the efforts of unskilled participants and, similarly to other efforts, the expectation might be that some level of non-compliance is expected. The desire, however, will be to implement the correct incentives such that the majority of efforts will be undertaken with the right focus and direction. Additionally, it will be important to consider what training or interventions might help unskilled students develop skills needed for STEGH, or how we might structure other STEGH to ensure that students are carefully embedded and supervised with an expectation to learn rather than lead. [1, 10, 21, 36]

Addressing these concerns and challenges will require critical elements of the strategy to deploy around international dialogues, host country resourcing and empowerment, and collaboration between various partners, such as institutions, regulatory colleges, and sending organizations.

## Conclusion

The deployment of a framework that targets societal, programmatic, and individual changes has long been effective in public health interventions such as tobacco control. Drawing on a parallel from these, STEGH is a growing and complex phenomenon with defined harms and identified challenges to their effectiveness and appropriateness. A comprehensive strategy that deploys both contextual and programmatic changes that will ultimately influence the conduct of such trips and change individual mindsets is essential.

While implementation of such a framework is challenging owing to resourcing, priority, and leadership considerations, the potential benefits from the strategies taken together in making STEGH more responsible, less harmful, and perhaps even a net positive to global health and development efforts should not be understated.
